# Antibacterial Potential of *Aloe weloensis* (Aloeacea) Leaf Latex against Gram-Positive and Gram-Negative Bacteria Strains

**DOI:** 10.1155/2019/5328238

**Published:** 2019-01-02

**Authors:** Yohannes Kelifa Emiru, Ebrahim Abdela Siraj, Tekleab Teka Teklehaimanot, Gedefaw Getnet Amare

**Affiliations:** ^1^School of Pharmacy, College of Medicine and Health Science, University of Gondar, Gondar, Ethiopia; ^2^School of Pharmacy, College of Medicine and Health Science, Wollo University, Dessie, Ethiopia

## Abstract

**Objective:**

To evaluate the antibacterial effects of the leaf latex of *Aloe weloensis* against infectious bacterial strains.

**Methods:**

The leaf latex of *A. weloensis* at different concentrations (400, 500, and 600 mg/ml) was evaluated for antibacterial activities using the disc diffusion method against some Gram-negative species such as *Escherichia coli* (ATCC 14700) and *Pseudomonas aeruginosa* (ATCC 35619) and Gram-positive such as *Staphylococcus aureus* (ATCC 50080) and *Enterococcus fecalis* (ATCC 4623).

**Results:**

The tested concentrations of the latex ranging between 400 and 600 mg·mL^−1^ showed significant antibacterial activity against bacterial strain. The highest dose (600 mg/ml) of *A. weloensis* leaf latex revealed the maximum activity (25.93 ± 0.066 inhibition zone) followed by the dose 500 mg/ml against *S. aureus.* The lowest antibacterial activity was observed by the concentration 400 mg/ml (5.03 ± 0.03) against *E. coli*.

**Conclusion:**

The results of the present investigation suggest that the leaf latex of *A. weloensis* can be used as potential leads to discover new drugs to control some bacterial infections.

## 1. Introduction

Infectious disease is defined as a disease caused by a specific infectious agent or its toxic product that results from transmission of pathogenic agent or its toxic product from an infected person, animal, or reservoir to a susceptible host [1]. Infectious diseases have been the most serious health issue in the world [2]. The epidemiology of infections has significantly changed in the last 30 years [3] and is responsible for one-third of global mortality [4]. Some of pathogenic infectious bacterial species includes *Staphylococcus aureus* (infections of skin, septic arthritis, and food intoxication) [5], *Bacillus subtilis* and *Bacillus licheniformis* (food intoxication) [6], *Esherichia coli* (food-borne illness, diarrhea) [7], *Pseudomonas aeruginosa* (infecting the chronic wounds) [8], and *Salmonella enterica* and *Salmonella typhimurium* (food intoxication) [9, 10]. To date, even though a wide range of synthetic and semisynthetic antibacterial medications are available for the treatment of infectious diseases [11], resistance of bacterial strains to the available antibiotic agents has been growing [12] and continues to challenge both developing and developed countries [13, 14]. Antimicrobial resistance is more frequent in low-income countries where one out of two people is dying prematurely from infections disease as compared to the developed countries [15, 16]. Moreover, the current cost of most of the chemotherapeutic agents is unaffordable to the patients that are especially found in developing countries [17]. As result of restricted access to proper medicine, in many developing countries, people are still using plants to treat the most prevalent infections [18].

The species *Aloe weloensis* belongs to the family Aloeacea. The leaf latex of *A. weloensis* has been used for the management of wound and different skin diseases and pain from ear infection, headache, and rheumatism in Ethiopian folk medicine [19]. Usage of plant-derived antimicrobial agents might provide opportunities to access new antibiotics and minimizing the chances resistance to pathogenic microorganisms [20]. Therefore, discovery of new-generation drugs against infections from natural products is highly desired for development of effective, affordable, and safe antibacterial agents that would be used as a complimentary or alternative with convectional medicines. To the best of our knowledge, there were no previous studies that have been conducted so far on the antibacterial activities of the leaf latex of *Aloe weloensis*. Hence, the objective of this study was to evaluate the antibacterial effects of the leaf latex of *Aloe weloensis* against infectious bacterial strains.

## 2. Methods

### 2.1. Collection and Identification of Plant

The leaf latex of *A. weloensis* was collected in the month of March 2018 from the local area (Harego, southeast of Dessie). The plant material was identified and authenticated by Prof. Sebsebe Demissew, Department of Biology, Addis Ababa University. A sample specimen of the plant has been deposited at the National Herbarium with a voucher number of TT-003.

### 2.2. Preparation of Latex

The latex of *A. weloensis* was collected by cutting the leaves transversally near the base and then latex from the leaves eluted by gravity in sterile plates by keeping the leaves at 45 to 90 degrees. The elution process was closely observed to avoid mixing of the latex with gel from the cut leaves. It was then left in open air for a period of 3 days to allow evaporation of water parts. Finally, after drying, a dark-purple color latex was obtained and stored in a refrigerator before screening for antibacterial activity.

### 2.3. Preparation of Inoculum

Each inoculum of standard bacteria strain was prepared by inoculating a loopful of test bacteria from a colony in 50 ml nutrient broth medium and mixed gently until it formed a homogenous suspension. The bacterial cultures were incubated at 37°C and grown to the mid-log growth phase. Finally, bacterial numbers were adjusted with 0.5 McFarland turbidometry.

### 2.4. Bacterial Strains

The four bacteria species which was used in this study were Gram-negative: *Escherichia coli* (ATCC 14700) and *Pseudomonas aeruginosa* (ATCC 35619) and Gram-positive: *Staphylococcus aureus* (ATCC 50080) and *Enterococcus fecalis* (ATCC 4623). All the standard bacterial strains were obtained from the Amhara regional laboratory.

### 2.5. Determination of Antibacterial Activity

Antibacterial activity of the leaf latex of *A. weloensis* (400, 500, and 600 mg/ml) was evaluated by the disc diffusion method [21]. In brief, the Mueller–Hinton agar (Himedia, Mumbai, India) plates were prepared by pouring into sterile Petri plates. The dried plates were swabbed uniformly with 0.1% of inoculum suspension and subsequently allowed to dry. The sterile individual discs loaded with various concentrations of the latex (400, 500, and 600 mg/ml) were placed onto the medium surface (pH 6.8–7.2). After diffusion of the latex into the medium, plates were incubated for 24 h at 37°C. For negative control, the discs were loaded with 10% dimethyl sulfoxide (Sigma-Aldrich, St. Louis, MO, USA) alone whereas ciprofloxacin (5 *μ*g/disc; Sigma-Aldrich, St. Louis, MO, USA) was used as positive control. After incubation, antibacterial activity was determined by measuring the zone of inhibition around the disc by millimeter using a transparent ruler. The measurements were performed in triplicates to determine the mean of the inhibition zone.

### 2.6. Phytochemical Screening

The phytochemical screening test of *A. weloensis* leaf latex was carried out for the presence or absence of secondary metabolites such as anthraquinolones, tannins, flavonoids, glycosides, alkaloids, and terpenoids by using the method as described by Trease and Evans [22] and Jones and Kinghorn [23].

### 2.7. Statistical Analysis

All the results were expressed as mean±standard error of means (SEM) for each microorganism. Statistical analysis was performed with the Statistical Package for the Social Sciences (SPSS) version 21.0. The differences between different concentrations were assessed by one-way analysis of variance (ANOVA). The post hoc multiple comparisons Duncan test were used. Results were considered significant when *p* ≤ 0.05.

## 3. Results

### 3.1. Antibacterial Activity of the Extract

*In vitro* antibacterial activity of the latex was studied against clinically important Gram-negative and Gram-positive strains of bacterial pathogens as presented in Table 1. The antibacterial activity was determined by the presence or absence of the inhibition zone around the discs. The tested concentrations of the latex ranging between 400 and 600 mg·mL^−1^ showed significant antibacterial activity against bacterial strain. The highest dose (600 mg/ml) of *A. weloensis* leaf latex revealed the maximum activity (25.93 ± 0.066 inhibition zone) followed by the dose 500 mg/ml against *S. aureus.* The lowest antibacterial activity was observed by the concentration 400 mg/ml (5.03 ± 0.03) against *E. coli*.

### 3.2. Phytochemical Screening

The results of Table 2 reporting the qualitative phytochemical analysis indicated the presence of all the tested bioactive compounds in the leaf latex of *A. weloensis.*

## 4. Discussion

Antimicrobial resistance is harmful to mankind because most of the infectious microorganisms understand the mechanism of drug action and develop tolerance to it [24]. Due to frequent development of resistance and possibility of the occurrence of harmful adverse effect up on the use of conventional antibacterial drugs [13], there is a continuous search to explore newer antibacterial agents with less side effect to the host from plant extracts effective against infectious microorganisms [11, 25].

For thousands of years, natural products have been used in traditional medicine all over the world and predate the introduction of antibiotics and other modern drugs [26]. In the present work, an attempt has been made to screening of *A. weloensis* leaf latex for its antibacterial activities against *S. aureus*, *E. coli*, *P. aeruginosa*, and *E. fecalis* by using the disc diffusion method. The antibacterial potential of *A. weloensis* latex was observed against tested bacterial strains in all concentrations.

Since, medicinal plants constitute a wide variety and diversity of secondary metabolites, medicinal plants could be used as a good source of antibacterial agents [27]. For instance, bioactive metabolites such as tannins and alkaloids [28], polyphenolic biomolecules [29], and flavonoids [30–32] have been found to have antimicrobial properties. The presence of such metabolites in the studied plant latex can provide a preliminary explanation on their antibacterial activities.

## 5. Conclusion

In conclusion, the current work demonstrated that the antibacterial activities revealed by *A. weloensis* were effective enough against Gram-positive and Gram-negative bacterial strains. The results presented in this study are encouraging and verify the search for newer active compounds in *A. weloensis* that is responsible for its antibacterial potential. Therefore, further antibacterial activity investigation of isolated compounds from the leaf latex of *A. weloensis* is highly warranted to pave the way to discover an efficient antibacterial agent that can be used alone or in combination with conventional antibiotics to treat infectious diseases caused by pathogenic bacterial strains and possibly to treat resistance bacterial strains.

## Figures and Tables

**Table 1:** Antibacterial activity of different concentrations of *Aloe weloensis* leaf latex.

Diameter of inhibition zone (mm)					
Microorganisms	Leaf latex (400 mg/ml)	Leaf latex (500 mg/ml)	Leaf latex (600 mg/ml)	DMSO (30 *µ*l/ml)	Ciprofloxacin (5 *μ*g/disc)
*S. aureus*	16.16 ± 0.16^a^	18.23 ± 0.12^a^	25.93 ± 0.066^a^	—^a^	27.03 ± 0.03^a^
*E. coli*	5.03 ± 0.03^a^	8.10 ± 0.10b^a^	10.10 ± 0.05^a^	—^a^	26.36 ± 0.18^a^
*P. aeruginosa*	12.13 ± 0.08^a^	14.06 ± 0.06^a^	11.06 ± 0.06^a^	—^a^	27.00 ± 0.00^a^
*E. fecalis*	15.10 ± 0.10^a^	17.03 ± 0.03^a^	14.16 ± 0.12^a^	—^a^	27.06 ± 0.03^a^

Results are given as mean ± SEM of ZI (excluded the diameter of the well) in mm (*n*=3). ^a^Statistical significance at *P* ≤ 0.05 (Duncan test); —, no zone of inhibition.

**Table 2:** Qualitative phytochemical constituents of *Aloe weloensis* leaf latex.

Class of compounds	Results
Anthraquinone	+++
Tannins	++
Flavonoids	+++
Glycosides	+++
Alkaloids	+++
Terpenoids	+

+, weak; ++, moderate; +++, strong.

## Data Availability

The datasets used and/or analyzed during the current study are available from the corresponding author on reasonable request.
